# Depletion of Regulatory T Cells in a Mouse Experimental Glioma Model through Anti-CD25 Treatment Results in the Infiltration of Non-Immunosuppressive Myeloid Cells in the Brain

**DOI:** 10.1155/2013/952469

**Published:** 2013-04-23

**Authors:** Wim Maes, Tina Verschuere, Anaïs Van Hoylandt, Louis Boon, Stefaan Van Gool

**Affiliations:** ^1^Laboratory for Thrombosis Research, Interdisciplinary Research Facility Life Sciences Kulak, E. Sabbelaan 53, 8500 Kortrijk, Belgium; ^2^Department of Neurosciences, Laboratory of Experimental Neurosurgery and Neuroanatomy, KU Leuven, Herestraat 49, ON1, Box 811, 3000 Leuven, Belgium; ^3^Laboratory of Pediatric Immunology, Department of Microbiology and Immunology, KU Leuven, Herestraat 49, ON1, Box 811, 3000 Leuven, Belgium; ^4^Bioceros B.V., Alexander Numan Building 2nd floor, Yalelaan 46, 3584 CM Utrecht, The Netherlands

## Abstract

The recruitment and activation of regulatory T cells (Tregs) in the micro-environment of malignant brain tumors has detrimental effects on antitumoral immune responses. Hence, local elimination of Tregs within the tumor micro-environment represents a highly valuable tool from both a fundamental and clinical perspective. In the syngeneic experimental GL261 murine glioma model, Tregs were prophylactically eliminated through treatment with PC61, an anti-CD25 mAb. This resulted in specific elimination of CD4+CD25hiFoxp3+ Treg within brain-infiltrating lymphocytes and complete protection against subsequent orthotopic GL261 tumor challenge. Interestingly, PC61-treated mice also showed a pronounced infiltration of CD11b+ myeloid cells in the brain. Phenotypically, these cells could not be considered as Gr-1+ myeloid-derived suppressor cells (MDSC) but were identified as F4/80+ macrophages and granulocytes.

## 1. Introduction

Escape from immunosurveillance has now been widely accepted as a hallmark of cancer. In early stages of malignancy, antitumor responses are mounted and are in many cases successful to eradicate the malignant cells. However, as malignancy progresses, few tumor cells escape the immunosurveillance, finally leading to clinically detectable tumors that are often very hard to cure [1]. This concept is also applicable in patients diagnosed with high-grade glioma. Malignant glioma cells have acquired a broad arsenal of strategies by which antitumor immunity can be countered and even reversed. Without any doubt, recruitment, expansion, and activation of Treg towards the tumor site is one of the dominant immunosuppressive mechanisms handled by glioma cells. Under physiological conditions, Tregs represent a final but crucial line of defence against the onset of autoimmunity caused by autoreactive T cells that have escaped the mechanisms of central tolerance in the thymus. The presence and detrimental contribution of Treg to antitumor immunity in the context of malignant glioma has been extensively documented both in clinical settings and in experimental models [2–7]. Furthermore, research in murine glioma models (such as the syngeneic GL261 model in C57BL/6 mice) recently focused on the development of strategies that allow (specific) elimination or silencing of tumor-induced Treg. In this perspective, low-dose cyclophosphamide treatment, CTLA-4 blockade, and STAT3 inhibition are promising [8–11]. We and others previously reported that a widely used rat monoclonal antibody (mAb), clone PC61, directed against the alpha chain of the mouse IL-2 receptor (CD25), which is highly expressed on natural Foxp3+ Treg, efficiently dampens their activity and restores the endogenous clearance of GL261 tumor cells by the immune system of the host mice [12, 13]. In the study presented here, local immunomonitoring revealed that prophylactic anti-CD25 treatment resulted in a pronounced infiltration of CD11b+ myeloid cells in the brain of glioma-bearing mice. Flow cytometric phenotyping revealed that these myeloid cells could not be classified as Gr-1+ MDSC but rather as F4/80+ macrophages and granulocytes. To our view, this is the first report describing that the depletion of Treg in an experimental (glioma) tumor model through PC61 treatment results in local infiltration of nonimmunosuppressive myeloid cells.

## 2. Materials and Methods

### 2.1. GL261 Brain Tumor Model

C57BL/6 mice were orthotopically challenged in the striatum with syngeneic GL261 cells or firefly luciferase- (Fluc-) transduced GL261 cells through stereotaxic surgery as previously described. Bioluminescence imaging was performed with an IVIS 100 system (Xenogen, Alameda, CA, USA) in the Small Animal Imaging Center of the KULeuven as described in [14]. All animal experiments were performed with permission of the Ethical Committee of the KULeuven on laboratory animal welfare.

### 2.2. Treatment with Anti-CD25 Monoclonal Antibody 

Three weeks prior to tumor challenge, 250 microgram of the anti-CD25 mAb clone PC61 (Bioceros B.V., Utrecht, The Netherlands) was administered intraperitoneally in a volume of 200 microliter sterile saline. Polyclonal rat IgG (Rockland, Gilbertsville, USA) was used as control.

### 2.3. Immunomonitoring

Brain-infiltrating immune cells were isolated as previously described [12]. Sorting of CD11b+ and CD11b− cells was performed using paramagnetic beads (Miltenyi Biotec, Bergisch Gladbach, Germany). Multicolor flow cytometry was performed using anti-mouse CD8a-FITC (53-6.7), CD4-PerCP-Cy5.5 (GK1.5), CD25-PE (7D4), Foxp3-APC (cFJK-16s), CD45− PerCP-Cy5.5 (30F11), CD11b-PE (M1/70), Ly6G/Gr-1-FITC (RB6-8C5), and F4/80-APC (BM8) mAbs (all from eBioscience, San Diego, CA, USA). For intracellular Foxp3 staining, the manufacturer's protocol was followed. For acquisition, a FacsCanto II flowcytometer (BD Biosciences, San Jose, CA, USA) was used, and data analysis was performed with FacsDiva software (BD).

Fluorescence minus one gating strategy was applied. Absolute cell numbers of each specific cell population were calculated by multiplying the relative cell numbers (as percent of total) with the total number of cells in a livegate. Cytospins were prepared on 2 × 10^5^ brain-infiltrating cells per sample by resuspending pelleted cells in 2 mL of sterile saline. The microscope slides were loaded with 500 microliter cell suspension, spun, and May-Grünwald Giemase stained. Analysis was performed with StereoInvestigator software (Microbrightfield, Magdeburg, Germany). For each sample, cell counts were performed in triplicates on a field of view (FOV) under 40x magnification.

### 2.4. Statistical Analysis

All data are represented as mean ± standard deviation. Nonparametric Mann-Whitney *U* testing was used. Statistics were calculated with Graphpad Prism software (Graphpad Software Inc., San Diego, CA, USA). In figures, ∗ and ∗∗, respectively, indicate *P* values of <0.05 and <0.01.

## 3. Results

### 3.1. Single-Dose Prophylactic Administration of Anti-CD25 Monoclonal Antibody PC61 Lowers CD4+CD25hiFoxP3+ Treg Infiltration in the Brain but Not CD4+CD25+Foxp3− and CD8+CD25−/+ Lymphocytes

**Figure 1:**
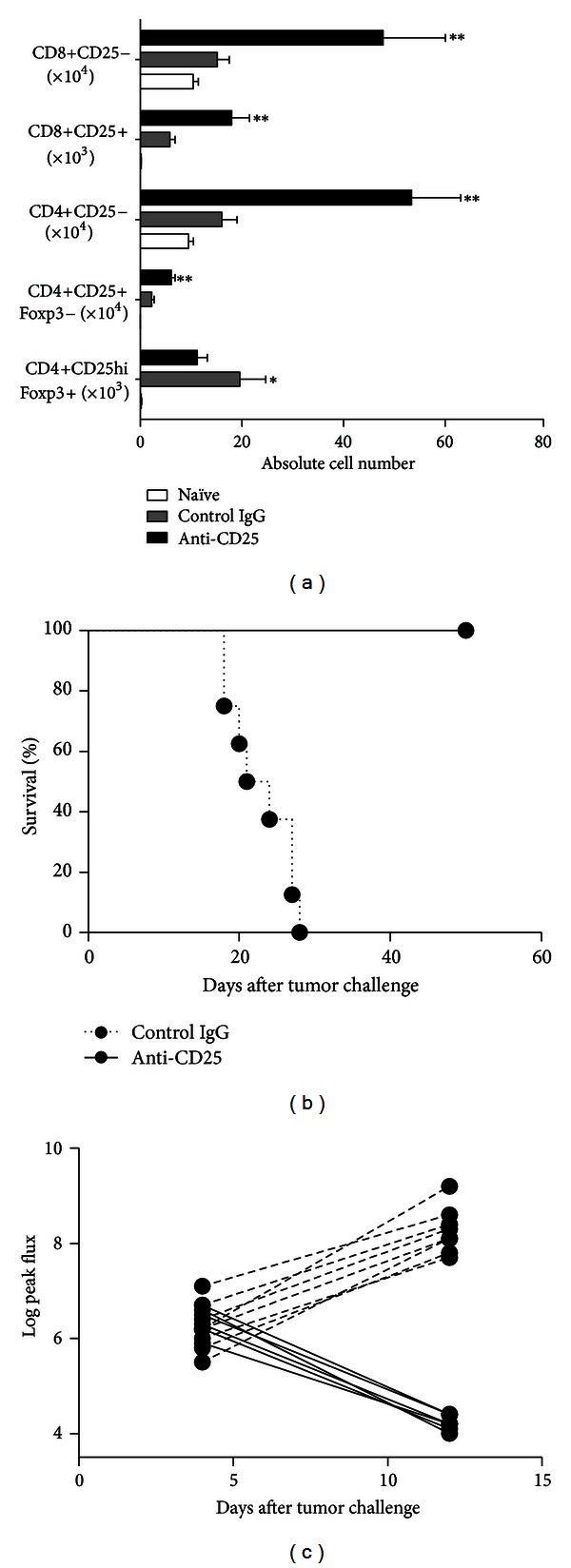
PC61 treatment specifically eliminates brain-infiltrating CD4+CD25hiFoxp3+ Treg. (a) Two weeks after tumor challenge, animals that were either prophylactically treated with PC61 anti-CD25 mAb (black bars, *n* = 11) or with control rat IgG (grey bars, *n* = 5) were sacrificed, and the brain-infiltrating CD4+ and CD8+ lymphocytes were analyzed by flow cytometry for expression of CD25 and Foxp3. Results are presented as absolute numbers. (b) Kaplan-Meier survival graph with PC61-treated mice (solid line, *n* = 9) and rat IgG control mice (dotted line, *n* = 8). Mice were tumor challenged on day 0. In one experiment, mice were challenged with Fluc-transduced GL261 cells. (c) Tumor burden was assessed through bioluminescence imaging on days 4 and 12 after tumor challenge of both PC61-treated mice (solid lines, *n* = 6) and control IgG-treated mice (dashed lines, *n* = 8). Data are presented as LOG (peak flux).

Treatment of C57BL/6 mice with a single dose of the anti-CD25 mAb clone PC61 prior to orthotopic glioma challenge significantly lowers the number of brain-infiltrating CD4+CD25hiFoxp3+ Treg as compared to IgG control animals when assessed 14 days after tumor challenge (11,2 ± 1,92 × 10^3^ *versus *19,6 ± 5,12 × 10^3^, *P* < 0.05). In contrast, the number of both CD4+CD25+Foxp3− cells (6,09 ± 0,76 × 10^4^ *versus *2,24 ± 0,50 × 10^4^, *P* < 0.01) and CD4+CD25− cells (53,4 ± 9,37 × 10^4^ *versus *16,1 ± 3,01 × 10^4^, *P* < 0,01) was significantly increased in PC61 treated mice. Likewise and irrespective of CD25 expression, CD8+ T cells were significantly increased (*P* < 0,01) in PC61-treated mice (Figure 1(a)). PC61-treated mice were completely protected against subsequent tumor challenge (survival up to 50 days after tumor challenge) compared to IgG-treated controls that displayed a median survival of 22 days (Figure 1(b)). To exclude the efficiency of the tumor challenges as variable, *in vivo *optical imaging was performed on mice that were challenged with Fluc-transduced GL261 cells, revealing that the initial tumor mass was comparable in all mice (Figure 1(c)). Furthermore, direct specific effects of the PC61 mAb on the GL261 tumor cells were excluded as expression of CD25 was absent on the GL261 glioma cells (data not shown).

### 3.2. Anti-CD25 Treatment Results in Local Infiltration of Nonimmunosuppressive Myeloid Cells in Glioma-Bearing Mice

**Figure 2:**
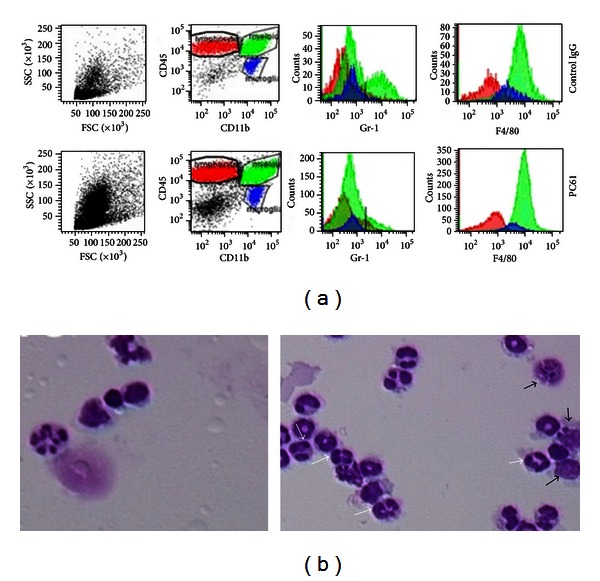
PC61 treatment of glioma-bearing mice leads to local infiltration with macrophages and granulocytes. (a) Flowcytometric phenotyping of brain-infiltrating myeloid cells in control (upper row) and PC61-treated mice (lower row). Based on the expression of CD45 and CD11b, three populations of brain-infiltrating cells were identified: CD45+CD11b− lymphocytes (in red), CD45+CD11b+ myeloid cells (in green), and CD45-CD11b+ microglial cells (in blue). Within the CD45+CD11b+ myeloid cells, expression of Ly6G (Gr-1) and F4/80 was measured. (b) Morphologic analysis of brain-infiltrating myeloid cells on cytospins. Detail from a representative FOV (40x) from control IgG-treated mice (left) and mice that were PC61 treated (right). Macrophages (black arrows) and granulocytes (white arrows) are highlighted.

**Figure 3:**
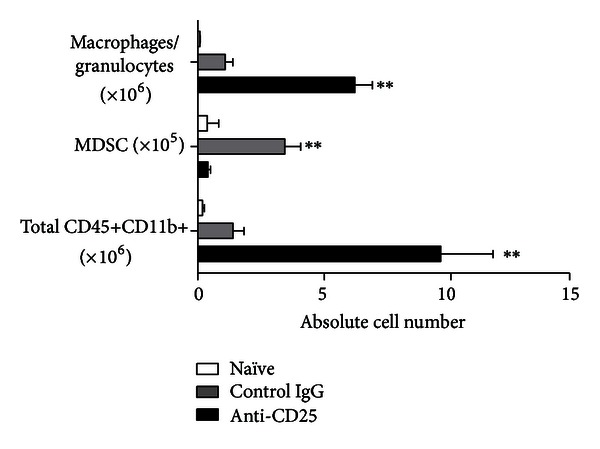
Quantitative analysis of myeloid cell infiltration in the brain of PC61-treated mice. Absolute numbers of brain-infiltrating total CD45+CD11b+ myeloid cells, Gr-1+ MDSC, and F4/80+ granulocytes/macrophages were calculated in PC61 (black bars, *n* = 9) and control IgG- (grey bars, *n* = 7) treated mice 14 days after tumor challenge. Naive mice were included as controls (white bars, *n* = 3).

PC61 treatment resulted in a pronounced increase (up to 3-fold more) of brain-infiltrating CD45+CD11b+ myeloid cells compared with IgG-treated control animals. Further characterization of this cell population was performed by analyzing the surface expression of Ly6G (Gr-1) and F4/80. In control mice, more Gr-1+ MDSCs were present within the CD45+CD11b+ cell population than in anti-CD25-treated mice (12.3 ± 2.8%  *versus *4.7 ± 1.6%, *P* < 0.05). The proportion of F4/80+ macrophages/granulocytes among CD45+CD11b+ myeloid cells was increased in PC61-treated mice (82.9 ± 3.53%) compared with IgG-treated mice (57.2 ± 8.23%, *P* < 0,05). A representative flowcytometric analysis for both PC61 and control IgG-treated mice is depicted in Figure 2(a). In naïve mice, both Gr-1+ MDSC and F4/80+ macrophages/granulocytes were virtually absent (data not shown). These results were confirmed by cell counting on cytospins revealing higher macrophage counts in PC61-treated mice (9.47 ± 0.95 cells/FOV, *P* < 0.01) compared with control IgG-treated mice (2.92 ± 0.38 cells/FOV). Similarly, when granulocytes were considered, PC61 treatment induced a significant higher influx (8.93 ± 1.35 cells/FOV, *P* < 0.01) compared with IgG-treated mice (4.42 ± 0.61 cells/FOV) (Figure 2(b)). Representative cytospin pictures are shown in Figure 2(b). In absolute numbers (Figure 3), PC61-treated mice harbored significantly more CD45+CD11b+ cells compared to control IgG-treated mice (9.60 ± 2.07 × 10^6^ *versus *1.39 ± 0.43 × 10^6^, *P* < 0.05). In control mice but not in PC61-treated mice, a higher influx of Gr-1+CD45+CD11b+ MDSC was noted compared to naïve animals (3.44 ± 0.63 × 10^5^ *versus *0.38 ± 0.09 × 10^5^, *P* < 0.05). PC61 treatment resulted in an increased infiltration of F4/80+CD45+CD11b+ macrophages/granulocytes compared to control animals (6.21 ± 0.69 × 10^6^ *versus *1.09 ± 0.30 × 10^6^, *P* < 0.01). In the brain of naïve mice, virtually no myeloid cells were detected.

## 4. Discussion

Together with others, we previously showed that treatment of mice with PC61, a widely used rat anti-mouse CD25 mAb, prolonged overall survival of tumor-bearing mice [15]. Moreover, the elimination of CD25hiFoxp3+ Tregs resulting from PC61 treatment was shown to be essential for effective vaccination with tumor lysate-pulsed dendritic cells and in combination with prophylactic DC vaccination, even resulted in the establishment of long-term immunity [12, 13].

In this study, we demonstrated that PC61 anti-CD25 mAb treatment of glioma bearing mice resulted in a decrease of brain-infiltrating Tregs only, whereas the CD4+ and CD8+ effector cells were increased and could exert their cytotoxic activity on the tumor cells. The complete protection of PC61-treated mice against tumor challenge underscores again the dominant immunosuppressive role of Treg in this model. Recently, Wainwright et al. reported that most of the brain-infiltrating Tregs in this model were FoxP3+ thymus-derived natural Treg [16]. 

To our view, this is the first report describing the marked infiltration of myeloid cells into the brain of GL261 tumor-bearing mice in which CD25 expressing Tregs were targeted with PC61. As was expected taking into account the beneficial effect on survival of PC61 treatment, we excluded that these cells were Gr-1+ MDSC [17]. On the contrary, PC61-treated mice even displayed less MDSC than control-treated mice. This could be explained by the fact that Tregs can induce or attract MDSC towards the tumor site (or vice versa), together building an immunosuppressive network [18]. In our hands, the brain-infiltrating myeloid cell pool consisted of both F4/80+ macrophages and granulocytes. Preliminary functional *in vitro *data reveal that these cells clearly exhibit phagocytic activity towards fluorescently labeled GL261 tumor cell lysate and dextran. A more in-depth analysis of *in vitro *antitumor activity of sorted CD45+CD11b+ cells would complete this picture. Moreover, Setiady et al. elegantly demonstrated that *in vivo *depletion of CD4+Foxp3+ Tregs through PC61 treatment is mediated through antibody-dependent-cellular phagocytosis by CD16+ (FcgammaRIII+) phagocytes (including both macrophages and granulocytes). In this perspective, it would be informative to run our model with PC61 treatment in FcgammaRIII^−/−^ mice [19]. Taken together, the data presented here further elucidate the local immunological events in the brain of GL261 tumor-bearing mice in which Tregs were targeted through treatment with anti-CD25 mAb PC61.

From a clinical perspective, Sampson et al. demonstrated in a pilot study that treatment with daclizumab, a humanized anti-CD25 therapeutic mAb, depletes Treg and correlates with enhanced immunity in glioblastoma patients [20]. Thus, Treg depletion in the context of glioma immunotherapy definitely warrants further investigation.
